# Language lateralization mapping (reversibly) masked by non-dominant focal epilepsy: a case report

**DOI:** 10.3389/fnhum.2023.1254779

**Published:** 2023-10-12

**Authors:** Belén Abarrategui, Valeria Mariani, Michele Rizzi, Luca Berta, Pina Scarpa, Flavia Maria Zauli, Silvia Squarza, Paola Banfi, Piergiorgio d’Orio, Francesco Cardinale, Maria Del Vecchio, Fausto Caruana, Pietro Avanzini, Ivana Sartori

**Affiliations:** ^1^“Claudio Munari” Epilepsy Surgery Center, ASST Grande Ospedale Metropolitano Niguarda, Milan, Italy; ^2^Department of Neurology, Hospital Universitario Puerta de Hierro, Majadahonda, Spain; ^3^Neurology and Stroke Unit, ASST Santi Paolo e Carlo, Presidio San Carlo Borromeo, Milan, Italy; ^4^Department of Neurosurgery, Fondazione IRCCS Istituto Neurologico Carlo Besta, Milan, Italy; ^5^Department of Medical Physics, ASST Grande Ospedale Metropolitano Niguarda, Milan, Italy; ^6^Cognitive Neuropsychology Centre, Department of Neuroscience, ASST Grande Ospedale Metropolitano Niguarda, Milan, Italy; ^7^Department of Biomedical and Clinical Sciences, Università degli Studi di Milano, Milan, Italy; ^8^Department of Philosophy “P. Martinetti”, Università degli Studi di Milano, Milan, Italy; ^9^Department of Neuroradiology, ASST Grande Ospedale Metropolitano Niguarda, Milan, Italy; ^10^Neurology and Stroke Unit, ASST Sette Laghi Ospedale di Circolo, Varese, Italy; ^11^Unit of Neuroscience, Department of Medicine and Surgery, Università degli Studi di Parma, Parma, Italy; ^12^Institute of Neuroscience, Consiglio Nazionale delle Ricerche, Parma, Italy

**Keywords:** epilepsy, case report, functional MRI, stereo-EEG, language mapping

## Abstract

Language lateralization in patients with focal epilepsy frequently diverges from the left-lateralized pattern that prevails in healthy right-handed people, but the mechanistic explanations are still a matter of debate. Here, we debate the complex interaction between focal epilepsy, language lateralization, and functional neuroimaging techniques by introducing the case of a right-handed patient with unaware focal seizures preceded by aphasia, in whom video-EEG and PET examination suggested the presence of focal cortical dysplasia in the right superior temporal gyrus, despite a normal structural MRI. The functional MRI for language was inconclusive, and the neuropsychological evaluation showed mild deficits in language functions. A bilateral stereo-EEG was proposed confirming the right superior temporal gyrus origin of seizures, revealing how ictal aphasia emerged only once seizures propagated to the left superior temporal gyrus and confirming, by cortical mapping, the left lateralization of the posterior language region. Stereo-EEG-guided radiofrequency thermocoagulations of the (right) focal cortical dysplasia not only reduced seizure frequency but led to the normalization of the neuropsychological assessment and the “restoring” of a classical left-lateralized functional MRI pattern of language. This representative case demonstrates that epileptiform activity in the superior temporal gyrus can interfere with the functioning of the contralateral homologous cortex and its associated network. In the case of presurgical evaluation in patients with epilepsy, this interference effect must be carefully taken into consideration. The multimodal language lateralization assessment reported for this patient further suggests the sensitivity of different explorations to this interference effect. Finally, the neuropsychological and functional MRI changes after thermocoagulations provide unique cues on the network pathophysiology of focal cortical dysplasia and the role of diverse techniques in indexing language lateralization in complex scenarios.

## 1. Introduction

In patients with focal epilepsy, brain language lateralization frequently diverges from the common left-lateralized pattern reported in healthy right-handed people. Indeed, right hemispheric dominance or bilateral patterns of either receptive, expressive, or both language aspects are reported in up to one-third of patients with focal epilepsy (Berl et al., 2014; Tailby et al., 2017), a prevalence 3–5 times higher than in healthy individuals. These “atypical patterns” are more frequent when epilepsy originates in the left hemisphere or seizure onset occurs at an early age (Dijkstra and Ferrier, 2013; Berl et al., 2014). Understanding language lateralization is clinically relevant, particularly in the presurgical assessment of drug-resistant epilepsies, to know the risk of damaging essential language regions when approaching the surgical removal of the epileptogenic zone (EZ). The occurrence of language impairment as a prominent feature of ictal semiology is considered a lateralizing sign, suggesting that the EZ is in the dominant hemisphere (Loesch et al., 2017).

Language assessment in epilepsy has evolved through a bunch of recording techniques, from the most traditional intracarotid amobarbital (Wada) test for language lateralization (Wada and Rasmussen, 2007). For language localization, the electrical stimulation mapping (ESM) introduced by Penfield and Roberts (1959) extensively spread in functional neurosurgery for intraoperative mapping (Ojemann et al., 1989) and also into the development of extraoperative cortical stimulation mapping, in parallel with the expansion of stereo-EEG and subdural grids for the presurgical evaluation of drug-resistant focal epilepsies (Hamberger et al., 2014; Trébuchon and Chauvel, 2016).

Neuroimaging techniques emerged in the last decades providing non-invasive methods (Schmid et al., 2018) for language lateralization, the more widespread being functional MRI (Bauer et al., 2014). Neuroimaging in epilepsy has been the basis for substantial research on the reorganization of language functions and networks. For instance, it has been proposed that the ongoing functional or slowly progressive structural disturbances due to epileptic activity might either shift language organization contralaterally (Janszky et al., 2006) or re-route language pathways to non-traditional areas within the dominant hemisphere (Deblieck et al., 2003; Liégeois et al., 2004).

Magnetic and electrophysiological recordings also contributed tools for the estimation of language lateralization (Salmelin, 2007; Ramon et al., 2009), especially thanks to the increasing density of the electrodes (Chu, 2015) and the development of source localization techniques capable of accurately reconstructing the intracortical generators (Lantz et al., 2003; Pascarella et al., 2023). These methods have been extensively used to localize the language network (Bowyer et al., 2020) as well as for reconstructing its temporal dynamics (Dalla Volta et al., 2018). In parallel, the high temporal resolution of EEG allows to quest for the identification of networks generating the interictal epileptiform activity (Avanzini et al., 2014; Tamilia et al., 2019), with benefits in the refinement of both diagnosis and treatment (Papadelis et al., 2022).

In the case of epilepsy, understanding the network between language organization and epileptic interictal/ictal discharges in the EZ would help to refine the estimation of language dominance, with fundamental implications for pre-surgical procedures and cognitive prognosis.

One missing piece of information is whether the effects of epilepsy on language organization are, to some extent, reversible once the epileptic activity is mitigated or removed. An ideal framework to tackle this issue is represented by stereo-EEG-guided radiofrequency thermocoagulations (RFTC), i.e., controlled focal lesions applied at the end of stereo-EEG monitoring to reduce the number and intensity of seizures (Cossu et al., 2015). This procedure not only reduces epileptic activity (Scholly et al., 2019) but also alters spontaneous activity through existing long-range patterns of connectivity (Russo et al., 2021).

Combining the potential of RFTC with the issue of language lateralization and organization in epileptic patients, two major questions remain unanswered. The first concerns whether RFTC can improve the functioning of language networks previously conditioned by epileptic activity arising from connected territories outside the language network. In parallel, an intriguing question is whether RFTC may also alter the estimation of language lateralization via neuroimaging techniques.

We address these issues by introducing the case of a patient with seizure-associated language impairment, thus suggesting epilepsy arising from the language-dominant hemisphere but presenting with a challenging definition of language lateralization. According to a neurological, neuroimaging, and neuropsychological assessment. Of note, functional MRI for language failed to indicate any clear lateralization. The subsequent stereo-EEG allowed identifying a right temporal EZ, propagating ictal activity to a left-dominant language network determining the language ictal disturbances. The EZ was thermocoagulated, and the patient showed improved language functions documented by neuropsychological tests. Notably, the functional MRI investigation repeated after the thermocoagulation revealed a clear left lateralization of language, indicating how epileptic activity can, in part reversibly, reduce the reliability of language lateralization by metabolic imaging.

## 2. Case report

A right-handed 31-year-old woman, a finance worker with drug-resistant focal epilepsy, was evaluated for epilepsy surgery in May 2017. Neurological examination was normal. From the age of five, she presented seizures that started with an acoustic illusion (“voices and noises overlapping in both ears”) accompanied by fear and warmth sensation, followed by an impairment of language (both comprehension and expression) and, subsequently, loss of awareness, and oroalimentary automatisms. After the seizure, language disturbance persisted for at least 10 min. She continued having weekly seizures.

**Figure 1:**
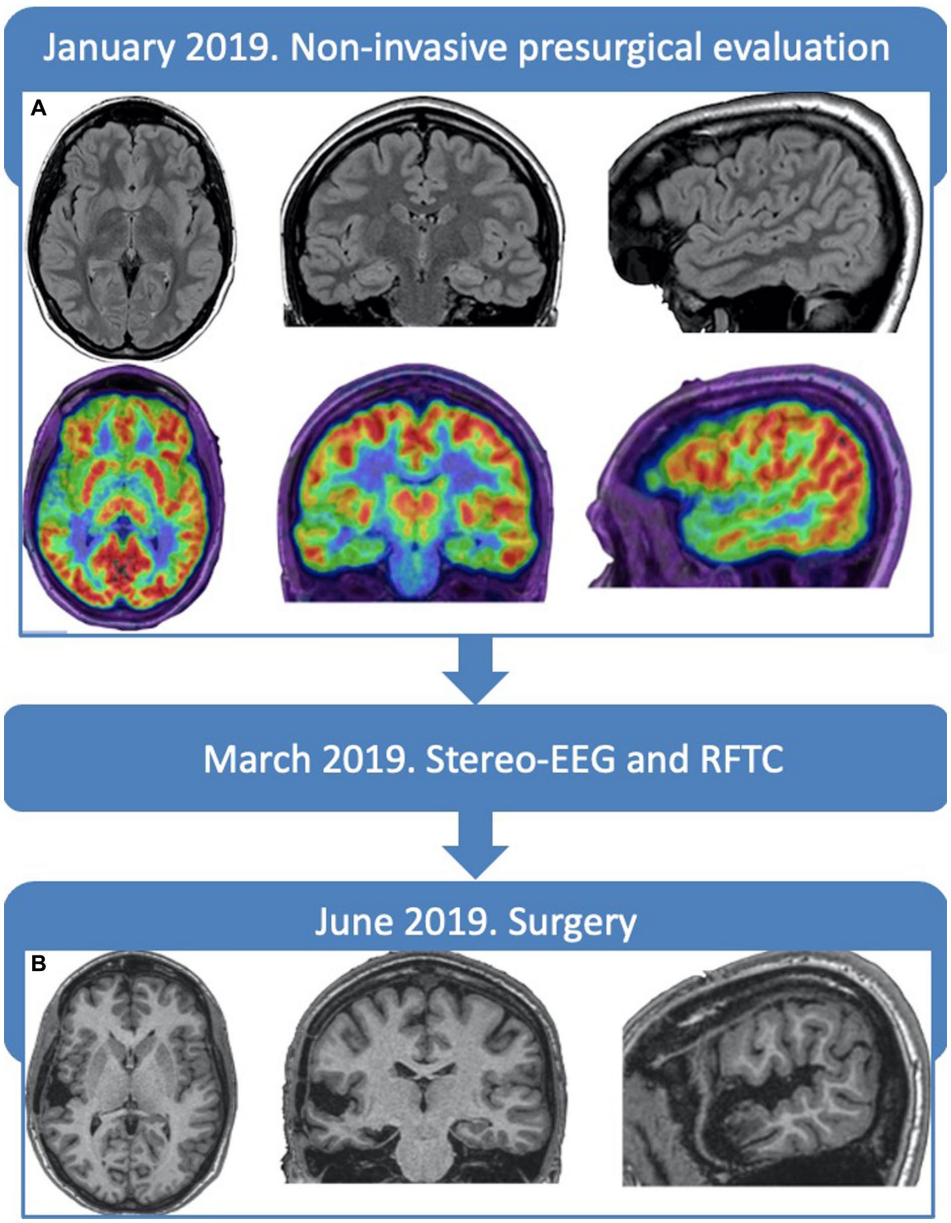
Timeline and neuroimaging. **(A)** Preoperative brain-MRI FLAIR slices, displaying no alterations and PET showing an hypometabolism of the right temporal lobe, more marked in the superior temporal gyrus and temporal pole. **(B)** Post-operative brain-MRI T13D slices, at 6 months, showing the resection cavity, without complications.

Prolonged video-EEG showed interictally bilateral asynchronous anterior and mid-temporal spikes. One seizure with usual semiology was recorded, suggesting an ictal onset in the right temporal region with early contralateral spreading. Brain MRI (1.5 Tesla) resulted normal, while a Positron Emission Tomography and Computed Tomography with 18F-fluorodeoxyglucose (18FDG-PET/CT) showed a right temporal hypometabolism, more marked at the level of the temporal pole and superior temporal gyrus (Figure 1A).

### 2.1. Non-invasive evaluation of language

The neuropsychological evaluation (Supplementary Table S1) showed a normal cognitive profile, except for a reduction in phonemic fluency (under the low limit of normal range compared to normative data obtained from an Italian population), naming (below the low limit), and verbal memory (at the inferior limit), a pattern suggestive of pathological involvement of the dominant hemisphere (Hermann et al., 1992).

A functional MRI was performed in an Achieva® 1.5 T magnet (Philips Healthcare, Best, The Netherlands). The acquisition consisted of a T2*-weighted gradient echo planar imaging sequence sensitive to blood oxygen level-dependent (BOLD) contrast. Three language paradigms- comprehension, association, and fluency - were acoustically administered (Silva and Citterio, 2017). The patient performed the tasks efficiently and had no seizures in the 15 days before the study. A trained specialist performed an unrestrained visual inspection of the whole-brain activity patterns assessing data quality and, in case of inconclusive contrasts, lowering the statistical threshold down to 0.05 uncorrected to verify whether lateralized patterns emerged.

**Figure 2:**
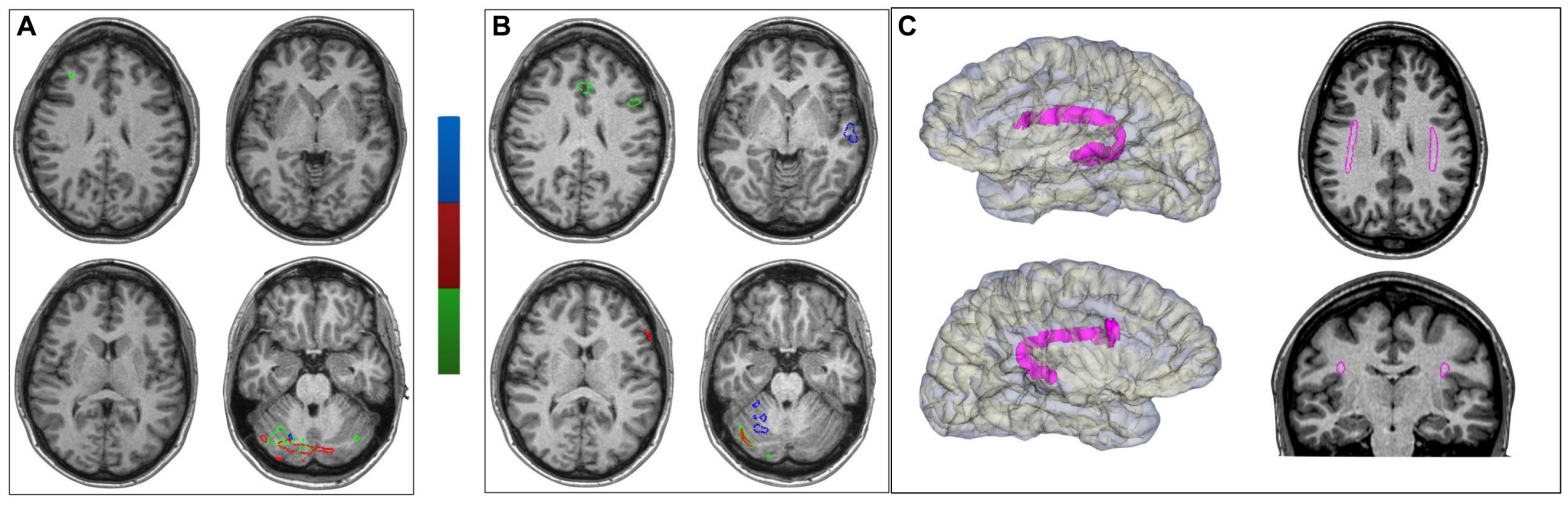
Functional MRI and DTI. **(A)** Functional MRI with language-dedicated tasks (phonemic and semantic fluency in green, auditory comprehension in blue and naming by verbal description in red). On the left side, the preoperative functional MRI showing a right cerebellar lateralization, with only a slight activation at the level of the right inferior frontal gyrus (the other two tasks were inconclusive). **(B)** The post-thermocoagulations functional MRI confirming the right cerebellar activation with left-side supratentorial activation either at the level of the inferior frontal gyrus for fluency and posterior temporal regions for comprehension. **(C)** Bilateral tractography representation of the superior longitudinal and arcuate fasciculi (SLF-III/AF) complex. On the left side: 3D surface rendering of each hemisphere and SLF-III/AF complex (in magenta). On the right side: axial and coronal slice of T13D brain-MR with representation of SLF-III/AF complex (in magenta). Left-side fasciculus volume resulted larger than the right-side one.

The functional MRI did not display any BOLD hemispheric activation during auditory comprehension and association tasks and only a slight activation of the right middle and inferior frontal gyrus during the fluency task. A right cerebellar activation was observed during all language tasks (Figure 2A).

Then, Diffusion Tensor Imaging (DTI) based fiber tracking was performed (Silva and Citterio, 2017). A probabilistic algorithm based on the “seed point-waypoint” approach was used, first identifying regions of interest (ROI) in the most frequent anatomical locations for the anterior and posterior language regions (Ojemann et al., 1989; Silva and Citterio, 2017). The white matter tracts were thus obtained using the posterior part of the superior temporal gyrus as the seed point and the anterior inferior frontal gyrus (pars triangularis and opercularis) as the waypoint. After the initial results in the two hemispheres, we reconstructed the “inter-hemispheric” connections by using the posterior ROI in the left hemisphere as the seed point and the posterior ROI in the right hemisphere as the waypoint.

The study showed the bilateral presence of a white matter tract connecting the inferior frontal gyrus and the superior temporal gyrus (belonging to the superior longitudinal and arcuate fasciculi systems), with asymmetry and a prevalence of the left-sided one (Figure 2C). These latter aspects have been reported as suggestive of left-sided language dominance (Glasser and Rilling, 2008).

### 2.2. Invasive evaluation of epilepsy

**Figure 3:**
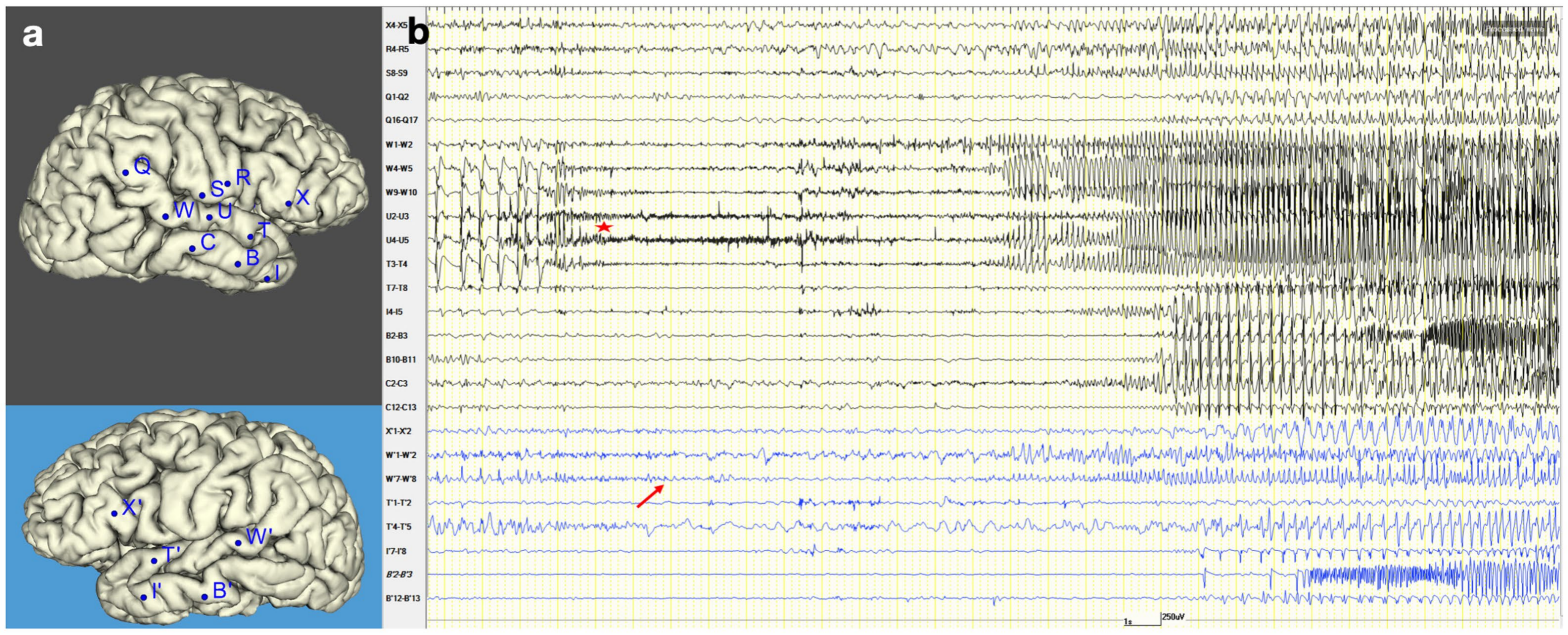
Seizure onset and propagation in stereo-EEG. **(A)** Stereo-EEG implantation: right hemisphere (black background) and left hemisphere (blue background). **(B)** Stereo-EEG recording of spontaneous seizure. It shows an increase of interictal spike on the contacts electrodes that explore the posterior portion of the right superior temporal gyrus (W4-5, W9-10, U4-5, T3-4), followed by low-voltage fast activity in the same region (red star). After 1.5 s, the discharge spreads to the left homologous region (W′7–8, red arrow). Then, after 14 s, it also involves the right temporal pole (I4-5) and hippocampus (C2-3), and immediately it propagates to the left homologous regions (I′7–8, B′2–3). After 26 s from the right ictal onset, a new ictal discharge arises in the left hippocampus (B′2–3) and spreads after 3 s into the right hippocampus (B2-3).

The inconclusive findings regarding the limits of a firmly suspected right temporal EZ made the patient eligible for an invasive study with intracerebral electrodes (stereo-EEG) (Cardinale et al., 2013, 2017). They were placed bilaterally (Figure 3) in tailored positions to identify the EZ and clarify language lateralization and its relationship with the EZ. The patient provided written informed consent. Stereo-EEG activity was continuously recorded through a 256-channel system (NIHON-KOHDEN NEUROFAX-1200). The sampling rate was 1,000 Hz. All acquisitions were referenced to two adjacent contacts located entirely in white matter.

The stereo-EEG interictal recording showed an altered background and continuous epileptiform discharges suggestive of type II focal cortical dysplasia (FCD) (Tassi et al., 2002) in the posterior portion of the right superior temporal gyrus, which proved to be also the location of the seizure onset. Seizures presented an early propagation to the left superior temporal gyrus that clinically coincided with the irruption of language disturbance. Further details on stereo-EEG recordings are reported in Figure 3.

### 2.3. Invasive evaluation of language

After recording spontaneous seizures on stereo-EEG, we proceeded to perform an ESM and analyze the gamma activity induced during language tasks.

On the left side, 50 Hz stimulations (0.5 ms pulse, 1 mA, 5 s train) in the posterior part of the superior temporal gyrus interrupted fluent speech, associated with an auditory illusion. In the same site, stimulations at 9 Hz (0.5 ms pulse, 5 mA, 15 s train) (Giovannelli et al., 2022) induced difficulty in expressive language and reading, confirming that site as a language hub. On the right superior temporal gyrus, low-frequency stimulations (1–3 Hz, 0.5 ms pulse, 5 mA, 15 s train) induced seizures similar to her spontaneous ones. On the left pars triangularis, 9 Hz stimulations induced a net slowing in reading, while no effect was evoked on the homologous right pars triangularis.

On a different day, we performed a “passive language mapping” by analyzing the gamma activity (50–300 Hz) (Arya et al., 2018; Trebuchon et al., 2020) induced by two computerized language tasks presented to the patient. The first one was a “listening” condition (240 brief sentences), and the second one was a “read-and-repeat” sequence (120 brief sentences). Data from leads were decomposed into time–frequency plots using complex Morlet’s wavelet decomposition. Thus, gamma power (50–300 Hz) was subdivided into non-overlapping 25-ms bins and estimated for adjacent 10-Hz frequency bands and normalized (*z*-score) to the interval [−500, 0 ms] before the stimulus presentation. Significance among homologous contacts located in the left and right inferior frontal gyrus, and separately among left and right superior temporal gyrus, were tested with a two-tailed paired t-test (*p* < 0.001) (Avanzini et al., 2016; Del Vecchio et al., 2019).

The gamma activation was significantly left lateralized in both the listening and the read-and-repeat condition, both in the superior temporal and inferior frontal gyrus. A remarkable activation during reading was observed in the left inferior frontal gyrus, congruent with the clinical slowing in reading observed during ESM (Figure 4.1).

**Figure 4:**
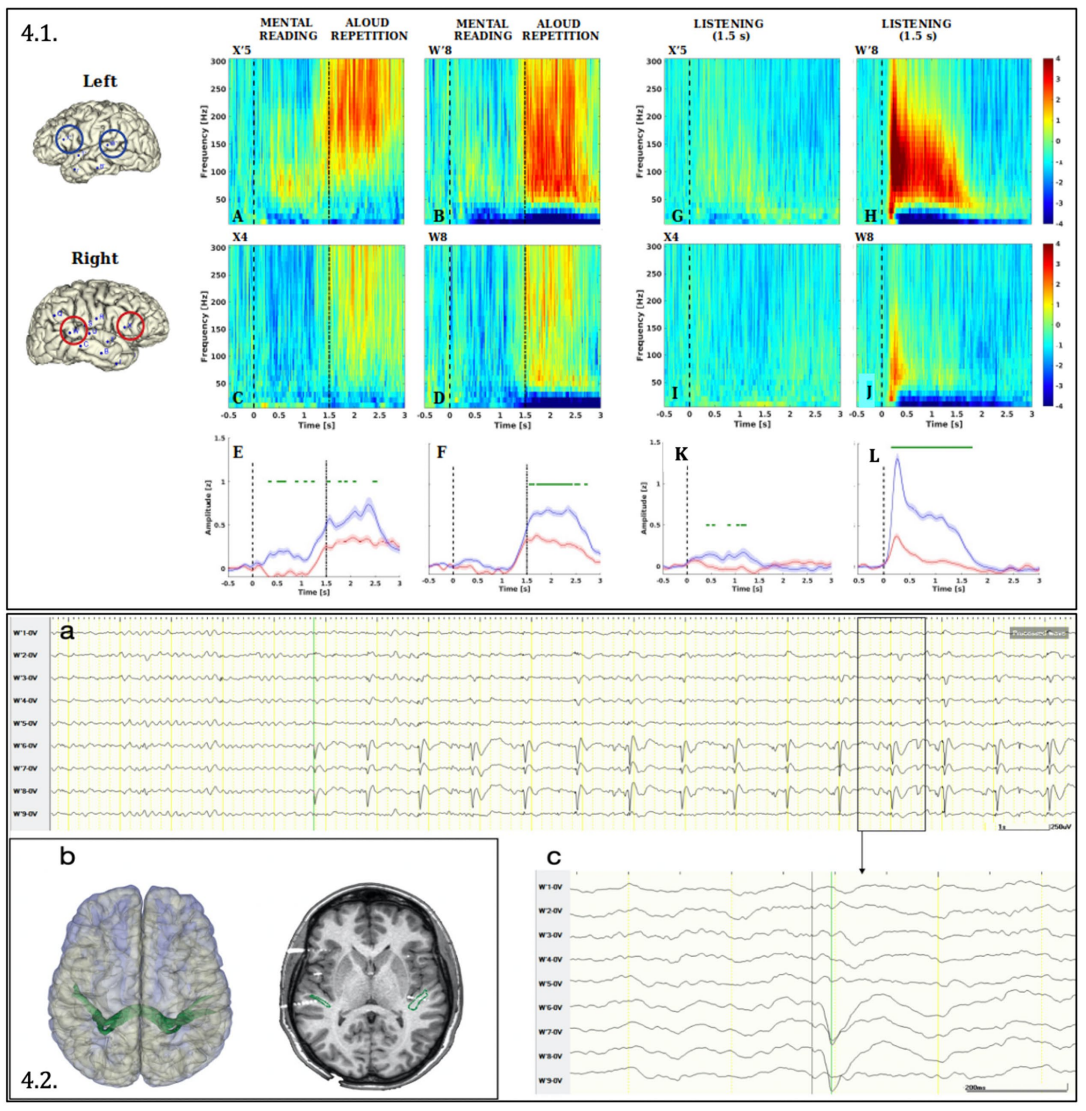
Analysis of Gamma activity (50–300 Hz) induced by language tasks **(4.1)** and Connectivity between the two homologous posterior temporal regions **(4.2.)**. **(4.1)** The Figure depicts the Event-Related Spectral Perturbation (ERPS) in the reading-repeat condition **(A-D)** and listening condition **(G–J)** for the leads sampling the cortex of the superior temporal gyrus (W′8 and W8) and inferior frontal gyrus (pars triangularis, X’5 and X4) of the left **(A,B,G,H)** and the right **(C,D,I,J)** hemisphere. **(E,F,K,L)** Compare for each couple of homologous leads the gamma-band (50–300 Hz) time course. Significance between left and right hemisphere has been computed with a paired, two-tailed *t*-test (*p* < 0.001) and plotted in green above the traces. Both data processing and statistical analysis were performed with Matlab R2022b and in-build function of EEGLAB package (Delorme and Makeig, 2004). ERSP (Delorme and Makeig, 2004) has been computed in the frequency interval (10–300 Hz) and baseline corrected (i.e., by subtracting the median in the interval [−500, 0 ms] according to the stimulus onset). **(4.2) (A)** Cortico-cortical evoked potentials obtained by means of stereo-EEG documented the connection between both posterior temporal regions: the stimulation (1 Hz, 0.5 ms, 5 mA) of the contacts W7-8 (right superior temporal gyrus) generated cortico-cortical evoked potential on contacts W′6; W′7; W′8 (left superior temporal gyrus) shown in monopolar montage. The latency between the stimulus and the evoked potential is 42 milliseconds (amplified below in **C**). **(B)** On the left side: 3D surface rendering of both cerebral hemispheres and the tractography-based fasciculus connecting both posterior temporal regions (in green). On the right side: co-registration between of preoperative 3D-T1 MRI, DTI-based “intertemporal fasciculus” representation and post-implantation cone beam CT, showing the intersection of the intertemporal fasciculus with W7-8 and W′6–7-8 contacts.

Cortico-cortical evoked potentials were visually analyzed using a low-frequency stimulation protocol (1 Hz, 5 mA, 0,5 ms pulse, 30 s train) applied through all electrode contacts (Matsumoto et al., 2017; Landré et al., 2018). They documented a connection between the inferior frontal and superior temporal gyrus in both the left and right hemispheres. Furthermore, they unveiled a bidirectional interhemispheric connection between the homologous regions of the superior temporal and inferior frontal gyrus. This last finding was confirmed also using tractography, which documented the interhemispheric structural connectivity among these areas (Figure 4.2 illustrates the connectivity between both superior temporal gyrus).

### 2.4. Treatment and outcome

The patient underwent stereo-EEG-guided RFTC applied through the electrode contacts exploring the posterior right superior temporal gyrus and the ipsilateral temporal pole. She remained seizure-free for 2 months. Then, seizures restarted with a lower frequency than before the procedure.

Three months after RFTC, a new neuropsychological evaluation revealed improved naming and verbal memory (up to normal scores) and stable phonemic fluency (Supplementary Table S1).

The functional MRI for language was repeated with the same protocol, revealing a left-hemispheric activation in all three tasks. On the contrary, right cerebellar activation remained similar to that unveiled during the preoperative exam (Figure 2B).

The EEG was performed 6 months after surgery and only slow activity in the right temporal derivations was present, in total absence of epileptiform abnormalities.

The patient was operated with a tailored resection within the limits of the right temporal lobe, including the superior gyrus, pole, and mesial structures (Figure 1B). She did not present any language disturbance in the post-surgical period. Histological examination revealed type IIb FCD in the posterior portion of the superior temporal gyrus. The patient is still seizure-free after 4 years and has withdrawn antiepileptic drugs.

One year after the resection, the neuropsychological evaluation showed increased phonemic fluency and confirmed the previously observed improvements (after RFTC) in verbal memory and naming (Supplementary Table S1).

## 3. Discussion

In the present study, we introduced the case of a patient whose epileptiform activity impacted language functions and the capacity of non-invasive explorations to identify her language dominance. The longitudinal and multimodal set of investigations conducted on the patient and the positive clinical outcome following stereo-EEG enabled us to document a complex interaction between epilepsy and the function of the language networks.

A right temporal origin of the seizures was firmly supported by video-EEG and 18FDG-PET/CT findings. However, this posed an issue with hemispheric language lateralization. Indeed, ictal aphasia could be explained by the propagation of seizures to a left-dominant hemisphere or by an “atypical” pattern of right hemispheric language lateralization. The neuropsychological evaluation supported the second hypothesis, as language and verbal memory impairments are more frequent in patients whose epilepsy originates in the dominant hemisphere (Hermann et al., 1992). The functional MRI was expected to clarify language lateralization, but the hemispheric BOLD activation was firstly unremarkable.

Different clinical conditions may lead to false-negative results during the functional MRI assessment of language lateralization. For instance, in tumor surgery, the weakness of the BOLD signal in regions adjacent to the tumor may lead to false negatives (Ulmer et al., 2004) due to impaired cerebrovascular reactivity. However, concerning patients with focal epilepsy, several studies reported an excess of atypical lateralization patterns (Tailby et al., 2017), yet whether epilepsy could hinder language lateralization in functional MRI examinations has been to date poorly investigated, with no evidence of an inter-hemispheric cross-talk. To our knowledge, this is the first case of false-negative or “masked” left-hemispheric activation driven by a contralateral FCD. Indeed, a previous single case study reported a temporary reduction of the left-hemisphere activation by language tasks after a cluster of left temporal seizures (Jayakar et al., 2002) but confined the effects to the same hemisphere.

In physiological conditions, neural metabolism and blood flow are tightly linked (“neurovascular coupling”). The increase in neural activity and metabolic demand when performing a task thus implies an increase in regional blood flow, oxygen supply, and subsequent BOLD signal in functional MRI (Weiss and Figueroa, 1998). On the contrary, both regional metabolism (measured by 18FDG-FET/CT) and blood flow (measured by Arterial Spin labeling MRI) are usually reduced at the level of an FCD, even beyond its boundaries (Chassoux et al., 2010; Blauwblomme et al., 2014); moreover, regional spontaneous BOLD fluctuations in resting-state functional MRI are also reduced (Gupta et al., 2018). Starting from these premises, a mechanistic explanation must be found for the surprising lack of functional MRI activation of language areas occurring contralaterally to the FCD and the site of PET hypometabolism. The more plausible hypothesis is that the FCD-generated epileptic discharges propagated directly from the right superior temporal gyrus, the site of the FCD, to the left superior temporal gyrus, one of the main nodes of the language network. The presence of such a connection was confirmed multimodally (DTI and CCEPs) in the patient. This right-to-left propagation of interictal activity would increase the level - and the variability - of basal BOLD signal not only in the posterior temporal regions but also the whole left-sided language network. As a result, in these regions the increase of metabolic demand induced by language tasks is not sufficient to reach the statistical significance. In other words, the left-sided activation during speech production would still take place, but on top of a basal signal that is larger and noisier than usual, *de facto* generating a false negative finding about cortical language lateralization.

An exceptional opportunity for causally testing this hypothesis came from repeating the functional MRI examinations and neuropsychological tests after the thermocoagulation of the FCD. Indeed, not only did the fMRI activation during language tasks recover the left-lateralized pattern coherent with the language dominance of the patient, but she also showed an improvement in the neuropsychological assessment. Starting from the previous observation that RFTC interferes with adjacent but also distant areas connected with the ablated cortex (Russo et al., 2021), we concluded that such intervention restored the proper functioning (and metabolic reactivity) of the language physiological network, previously interfered with by the epileptic activity originating in the non-dominant hemisphere. These results align with the notion that neural plasticity involves local synapses in the cortex and their related networks (Pasquini et al., 2022).

The case described here provides valuable insights for the non-invasive study of language lateralization in daily clinical practice. We propose that language lateralization in epileptic patients cannot be investigated without accounting for the topography of the EZ and the propagation of epileptic activity, which could alter or mask the language-related contrast. Beyond using EEG-fMRI approaches that model the interictal epileptiform discharges into the functional MRI signal analysis (Gotman, 2008), two parallel tools of MRI can reinforce the estimation of language dominance in epileptic patients.

The first derives from the (right) cerebellar functional MRI activation stability across all the language tasks in pre- and post-thermocoagulation recordings. The right cerebellar hemisphere plays a role in different language processing components through crossed cerebro-cerebellar connections (Stoodley and Schmahmann, 2009). Likewise, healthy subjects with right-brain language hemispheric lateralization show left cerebellar activation in language functional MRI studies (Jansen et al., 2005; Vias and Dick, 2017). Epilepsy literature has rarely paid attention to this point, with only one pediatric epilepsy study concluding that crossed cerebro-cerebellar language activation may be a key feature of language networks that remains preserved even against massive reorganization due to cerebral lesions or epilepsy (Gelinas et al., 2014). Indeed, in our patient, the cerebellar BOLD activation correctly identified language lateralization even in a context of masked cerebral hemispheric activation.

Further, asymmetry of the superior longitudinal/arcuate fasciculi systems emerged as another potential tool for revealing language lateralization when functional MRI data are ambiguous through a bilateral evaluation of symmetry and volume (Glasser and Rilling, 2008; Silva and Citterio, 2017; Bain et al., 2019)^.^ In the present case, this asymmetry was more informative than the initial functional MRI lateralization, suggesting that the alterations driven by epileptic activity are larger at the functional level (cortical BOLD reactivity) than the structural one (diffusion tractography). Although previous studies have proposed a coincidence between grades of lateralization of BOLD activations in functional MRI and features of the arcuate bundle in DTI, the coincidence in patients whose language in functional MRI is not left-lateralized, and the underlying mechanistic explanation, is still a matter of debate (Vernooij et al., 2007; Di Cristofori et al., 2021; Verhelst et al., 2021).

Primary for the propagation of epileptic activity to the left hemisphere was an interhemispheric connection between both superior temporal gyri that has been scarcely studied in previous literature (Umeoka et al., 2009; Wei et al., 2017). This connectivity was demonstrated by tractography, cortico-cortical evoked potentials (Figure 4.2), and the stereo-EEG recording of the right-to-left ictal spreading (Figure 3). It is unclear whether this connection is present physiologically or only in the context of congenital lesions (as in the present case). It could also be a “rescue option” after an insult at the level of language areas: functional MRI studies revealed that homologous contralateral regions are recruited in the early phase following a perisylvian stroke (Saur et al., 2006). In physiological conditions, these connections are probably masked by the transcallosal interhemispheric inhibition, which decreases after this type of damage (Hamilton et al., 2011).

## 4. Conclusion

Patients with epilepsy and language disturbances need particular attention when interpreting non-invasive studies of language lateralization. Our case demonstrates that the physiological activation of left hemisphere language regions can be disturbed by the epileptiform activity of an FCD located contralaterally but in a region anatomo-functionally connected with the left-sided language network. As continuous epileptiform activity (like that arising from FCD) may significantly affect cortical activation on functional MRI, the etiology of epilepsy must be considered during the investigation of language lateralization. In case of inconclusive functional MRI cortical contrasts, we suggest incorporating in clinical practice the quantification of cerebellar activations and arcuate fasciculus asymmetry by DTI.

## Data availability statement

The original contributions presented in the study are included in the article/Supplementary material, further inquiries can be directed to the corresponding author.

## Ethics statement

The studies involving humans were approved by A.S.S.T. Grande Ospedale Metropolitano Niguarda. The studies were conducted in accordance with the local legislation and institutional requirements. The participants provided their written informed consent to participate in this study. Written informed consent was obtained from the individual(s) for the publication of any potentially identifiable images or data included in this article. Written informed consent was obtained from the participant/patient(s) for the publication of this case report.

## Author contributions

BA: Conceptualization, Writing – original draft, Writing – review & editing. VM: Conceptualization, Writing – original draft, Writing – review & editing. MR: Formal analysis, Software, Writing – original draft, Investigation. LB: Formal analysis, Software, Writing – original draft, Investigation. PS: Methodology, Writing – original draft, Investigation. FZ: Data curation, Formal analysis, Writing – original draft, Investigation. SS: Formal analysis, Writing – original draft, Investigation. PB: Resources, Writing – original draft, Investigation. Pd’O: Formal analysis, Software, Writing – original draft, Investigation. FrC: Software, Writing – original draft, Investigation. MD: Software, Writing – original draft, Formal analysis, Investigation. FaC: Writing – review & editing, Investigation. PA: Funding acquisition, Methodology, Supervision, Writing – original draft, Writing – review & editing. IS: Supervision, Writing – original draft, Writing – review & editing, Methodology.

## Abbreviations

BOLD: blood oxygen level-dependent
DTI: diffusion tensor imaging
EZ: epileptogenic zone
ESM: electrical stimulation mapping
FCD: focal cortical dysplasia
RFTC: radiofrequency thermocoagulations
ROI: regions of interest
18FDG-PET/CT: Positron Emission Tomography and Computed Tomography with 18F-fluorodeoxyglucose
