# Supplementary material for: Language lateralization mapping (reversibly) masked by non-dominant focal epilepsy: a case report
Source: Front Hum Neurosci. 2023 Oct 12;17:1254779. doi: 10.3389/fnhum.2023.1254779 (PMC10600519; doi:10.3389/fnhum.2023.1254779)
Supplement: Supplementary file 1 [file Table_1.DOCX]

**Table 1 Neuropsychological evaluation (memory and language domains).**

| **Evaluated Functions and test** | Reference value | **Basal evaluation (20/3/2019)** | | **After stereo-EEG (19/6/2019)** | | **1 year after surgery (23/9/2020)** | |
| --- | --- | --- | --- | --- | --- | --- | --- |
|  |  | Raw scores | Adjusted scores | Raw scores | Adjusted scores | Raw scores | Adjusted scores |
| **Language** |  |  |  |  |  |  |  |
| Phonemic fluency |  | 19 | 0* | 20 | 0* | 27 | 1 |
| Semantic fluency |  | 47 | 3-4 | 38 | 2 | 34 | 1 |
| Naming | 24 | 20* |  | 24 |  | 24 |  |
| Token Test |  | 33 | 2 | 33 | 2 | 35 | 3 |
| **Verbal memory** |  |  |  |  |  |  |  |
| Digit span forward |  | 5 | 2 | 5 | 2 | 6 | 3 |
| Digit span backward |  | 3 |  | 3" |  | 5 | 3 |
| Verbal paired associates |  | 14 | 3 | 17 | 4 | 16.5 | 4 |
| Short story |  | 14 | 1 | 16 | 2 | 12.5 | 1 |
| Immediate recall |  | 14 |  | 15 |  | 9 |  |
| Delayed recall |  | 14 |  | 17 |  | 16 |  |
| **Visuo-spatial memory and visuo-costructive skills** |  |  |  |  |  |  |  |
| Corsi Cubes span |  | 6 | 4 | 6 | 4 | 7 | 4 |
| Corsi Supraspan |  | 24.75 | 3 | 23.65 | 3 | 27.18 | 4 |
| Rey Figure: |  |  |  |  |  |  |  |
| copy | 32 +/- 18 | 34 |  | 30 |  | 32 | 1 |
| recall | 22 +/- 4.9 | 17 |  | 20 |  | 20 | 4 |
| Camden Short Recogn. Memory Test | 22.9 +/- 2 | 24 |  |  |  | 24 |  |
| All values without any symbol are within the normal range.; scores under the normal range are pointed with a symbol *. | | | | | | | |
| Adjusted scores are calculated comparing to normative data obtained from italian population: 0, under inferior limit of normal range; 1, inferior limit of normal range; 2-3, normal range; 4, over 50 percentil. | | | | | | | |
| For test without available normative data, the reference value is specified in the first column. | | | | | | | |
